# Could Contamination Avoidance Be an Endpoint That Protects the Environment? An Overview on How Species Respond to Copper, Glyphosate, and Silver Nanoparticles

**DOI:** 10.3390/toxics9110301

**Published:** 2021-11-11

**Authors:** M. Antonella Alcívar, Marta Sendra, Daniel C. V. R. Silva, Enrique González-Ortegón, Julián Blasco, Ignacio Moreno-Garrido, Cristiano V. M. Araújo

**Affiliations:** 1 Department of Agricultural Chemistry and Soil Science, University of Cordoba, 14071 Córdoba, Spain; antonellaalcivar25@gmail.com; 2 Institute of Marine Research (IIM), Spanish National Research Council (CSIC), Eduardo Cabello 6, 36208 Vigo, Spain; msendra@iim.csic.es; 3 Institute of Exact Sciences, Federal University of Southern and Southeastern Pará, Marabá 68507-590, Pará, Brazil; daniel_cruzeiro@yahoo.com.br; 4 Department of Ecology and Coastal Management, Institute of Marine Sciences of Andalusia (ICMAN), Campus Río San Pedro, Puerto Real, 11510 Cádiz, Spain; e.gonzalez.ortegon@csic.es (E.G.-O.); julian.blasco@csic.es (J.B.); ignacio.moreno@icman.csic.es (I.M.-G.)

**Keywords:** environmental heterogeneity, multi-compartment exposure system, non-forced exposure, sensitive profile, species sensitivity distribution

## Abstract

The use of non-forced multi-compartmented exposure systems has gained importance in the assessment of the contamination-driven spatial avoidance response. This new paradigm of exposure makes it possible to assess how contaminants fragment habitats, interfering in the spatial distribution and species’ habitat selection processes. In this approach, organisms are exposed to a chemically heterogeneous scenario (a gradient or patches of contamination) and the response is focused on identifying the contamination levels considered aversive for organisms. Despite the interesting results that have been recently published, the use of this approach in ecotoxicological risk studies is still incipient. The current review aims to show the sensitivity of spatial avoidance in non-forced exposure systems in comparison with the traditional endpoints used in ecotoxicology under forced exposure. To do this, we have used the sensitivity profile by biological groups (SPBG) to offer an overview of the highly sensitive biological groups and the species sensitive distribution (SSD) to estimate the hazard concentration for 5% of the species (HC_5_). Three chemically different compounds were selected for this review: copper, glyphosate, and Ag-NPs. The results show that contamination-driven spatial avoidance is a very sensitive endpoint that could be integrated as a complementary tool to ecotoxicological studies in order to provide an overview of the level of repellence of contaminants. This repellence is a clear example of how contamination might fragment ecosystems, prevent connectivity among populations and condition the distribution of biodiversity.

## 1. Ecotoxicology and Avoidance in a Chemically Heterogeneous Landscape

During the last 50 years since ecotoxicology was proposed as a new science [1], there has been a continuous advance regarding the number of methods, test species, and responses employed to assess the effects of contamination on organisms and ecosystems. The search for the most sensitive species has led researchers to test numerous species from different biological groups, trophic levels, and geographic distribution [2,3]. However, the concept of the most sensitive species has become obsolete as it is a rather theoretical concept since one species can be very sensitive to a given class of contaminants, but less sensitive to another one [2,4,5,6]. Alongside the need to standardize the test procedures adopted by industries and governments as a legal tool for the environmental risk assessments (ERAs) conducted; researchers need to use organisms that meet some basic requisites besides the sensitivity and ecological relevance [7]. For example, it should be relatively easy and cheap to culture organisms in a laboratory and the procedures to test toxicity should be simple and practical [3]. For this reason, ecotoxicology focused particularly on the growth inhibition tests with microalgae, and mortality/immobilization and reproduction tests with daphnids and fish. Over the years, organisms from temperate zones (mainly from Europe and United States) have been used widely, regardless of their importance for other ecosystems, because the experimental procedures were technically more developed and standardized [8,9]. However, many researchers from different geographic areas turned their focus to local key species, adapting or even creating new experimental procedures for the species considered of ecological importance. All these processes coupled with the rapid development of molecular biology (from the perspective of the sub-individual; [10]) and to higher integration of ecological concepts (from a perspective of ecosystem structure and functioning; [11]), have favored unprecedented advances in the field of ecotoxicology. Today, ecotoxicological studies are able to provide valuable information about the risk contaminants represent to organisms, although some limitations still exist regarding the extrapolation to natural ecosystems [12].

In this context, one classical paradigm of the ecotoxicity tests is the continuous and mandatory exposure of organisms to contaminants. Those tests assume that organisms in natural ecosystems are forcedly exposed to contaminants, with no possibility of fleeing. However, complementary methods, in which organisms are simultaneously exposed to several concentrations and can choose the most favorable one, have been proposed (see review by Jutfelt et al. [13]). Initially, these methods provided a bi-compartmentalization of the system (with and without contaminant), but with the limitation of not allowing the calculation of AC*x* (the concentration that triggers the avoidance of x% of the population) that is analog to the classical LC*x* (lethal concentration), EC*x* (effective concentration), etc. That, to some extent, prevents the comparison of data obtained from both approaches. However, new methods using non-forced, multi-compartmented linear exposure systems (linear 1-D system by Lopes et al. [14] and 2-D HeMHAS by Araújo et al. [15]) have been developed recently. The main benefit of the multi-compartmentalization exposure systems is the possibility of determining the concentrations of a contaminant in each zone (compartment) through which the organisms can move freely, providing an idea of the potential repellence or attractiveness of the contaminants [14,16,17]. It is important to bear in mind that this approach should be seen as a complementary tool to the classical forced exposure approach, as the non-forced approach provides information about how contamination could affect the spatial distribution of the organisms, but not about the toxic effects [17,18]. Thus, the concept of toxicity at the individual level is replaced by the effects on the dynamics of dispersion (spatial avoidance) and habitat selection, from a landscape (connected habitats) perspective [19,20,21]. Although non-forced exposure supposes no effect at the individual level, the fleeing of a species from an ecosystem could, ecologically, be considered similar to the death of the individuals [14]. Due to this methodological and conceptual particularity of the non-forced multi-compartmented approach, an important question arises: how sensitive is the avoidance response in highlighting the potential risk of a chemical compound?

Although the amount of data generated by ecotoxicology has been considerable over the last few years for many contaminants, especially the contaminants of emerging concern (new agrochemicals, nanoparticles, sunscreens, pharmaceutical products, plastic derivatives, etc.; [22]), information is still scarce. This seems to be highlighted when a new paradigm such as the non-forced multi-compartmented exposure is to be applied. Although this exposure approach has increased in ecotoxicological studies (see reviews by Araújo et al. [23] and Moreira-Santos et al. [24]), information about the real potential of contaminants to trigger avoidance in organisms and to change their habitat selection patterns is very limited. In addition, it is not clear whether toxicity and repellency are comparable in terms of sensitivity [17].

The current review aims to assess how sensitive the avoidance response measured in multi-compartmented exposure systems is in comparison with the various toxic responses used in ecotoxicology from forced exposure experiments. To this end, a sensitivity profile by biological groups (SPBG; [25] for three reference contaminants (copper, glyphosate, and silver nanoparticles—Ag-NPs) was created. The SPBG is a simple way to identify the biological groups that could be considered more susceptible and the groups of responses that could provide an idea about the main toxic effects expected to occur. Secondly, we assessed whether the concentrations that trigger an avoidance response for 50% of the population (AC_50_) will be among the responses that are expected to occur at concentrations considered hazardous for 5% of the species (HC_5_; [6]). Finally, we discuss: (i) the sensitivity of the avoidance response as an endpoint (focusing on the repellence of contaminants) from non-forced exposure approaches compared to toxicity data from forced exposure, (ii) the feasibility of using the avoidance response in multi-compartmented systems as a complementary tool in ERAs, and (iii) the ecological relevance and improvements that could result from integrating the avoidance response into ecotoxicological studies.

## 2. Chemicals Used as Reference Contaminants

To compare the sensitivities among avoidance response and other endpoints, three chemicals with completely different chemical characteristics and modes of action were chosen as the reference contaminants: copper, glyphosate, and Ag-NPs. Copper was selected as one of the most traditional chemical compounds used in ecotoxicology [26,27] with an ample amount of data available and because it is one of the most ubiquitous contaminants used in different sectors such as industry and agriculture. Glyphosate was also selected because it is one of the most widely used pesticides in the world and it is the object of widespread controversy concerning the effects it can produce on non-target organisms [28,29]. Finally, Ag-NPs are a contaminant of emerging concern chosen due to being one of the most common nanomaterials found in consumer products such as antimicrobial agents [30,31]. More than 100 results of ecotoxicological data were revised and included in the current study for each contaminant (Tables S1–S3). Particularly in the case of Ag-NPs, avoidance experiments were performed in multi-compartmented systems to compensate for the absence of data in the literature and make it possible to compare the results. The experiments are described briefly in the next section.

## 3. Avoidance Assays with Ag-NPs

Ag-NPs (<15 nm in aqueous suspension; US7140—US Research Nanomaterials, Inc., Houston, TX USA) described by Sendra et al. [32] were used. Avoidance assays were performed in the non-forced, six-compartmented exposure systems used by Islam et al. [33], and zebrafish (*Danio rerio*) were used as the test organisms. Initially, different concentrations of Ag-NPs (0, 5, 10, 20, 40, and 80 µg/L) were prepared and put into the system in the form of a gradient. Afterward, five juveniles of zebrafish (body size: 2.0 to 2.5 cm) were introduced in each concentration; therefore, 30 organisms were used in each replicate. The experiment was run in triplicate. The displacement of the fish was recorded at different time intervals: 30, 60, 90, 120, 150, and 180 min and after 24 h. A red light was used during observation to minimize the interference of the observer on the behavior of the fish. Exposure was performed at 22 °C and in the dark. More details about the assay are described in Islam et al. [33].

## 4. Sensitivity Profile by Biological Groups: Definition

SPBG is a representation of the potential toxicity of contaminants intended to provide information on the sensitivity of the ecotoxicological responses measured in different biological groups. This representation offers an overview that could help researchers to better identify the biological groups and the endpoints that could be more suitable to assess the toxicity of a specific, or class of, chemical (s). This representation was chosen because it provides a clear visual panorama of how sensitive the avoidance response might be when compared with other endpoints. All the information of the studies analyzed can be verified in Tables S1–S3.

The data collected to create the sensitivity profile were based on the concentrations that cause 50% of effect (EC_50_). Although NOEC (the highest no-observed effective concentration) or LOEC (the lowest observed effective concentration) could be more protective environmentally, they were not chosen because both are dependent on the concentrations used in the studies. The use of the EC_50_ values did not have an environmental criterion, but instead, it was adopted to standardize the database.

The first step to creating the SPBG consisted in revising published papers looking for different species and different responses to create a sufficiently robust and diverse (as many species and responses as possible) database. The review of the literature (Google Scholar, Scielo, Scopus, and Web of Knowledge) was performed using various words like aquatic, ecosystems, EC or LC or IC, sensitivity, toxicity, and the name of the contaminant (copper, glyphosate, or Ag-nanoparticle). No selection about the year was made. Preferably, studies published in high impact factor journals (Q1 and Q2 according to JCR index) were selected, except if the study presented particular data for a determined species or response. When an imbalance regarding the amount of data for a given biological group or endpoint was identified, a more specific search taking into consideration the response or biological group of interest was performed, to provide more information about the specific biological group or endpoint. All the species were classified by biological groups in accordance with the groups described in Table 1 (see also Tables S1–S3). When the number of species in a group in the database was very high (like Crustacea), different subgroups (such as cladocerans, shrimps, crabs…) were created to discriminate any differences in the sensitivity of each subgroup. Data were not separated by species because the species used in a geographic region are not necessarily representative of another region; therefore, the organization by biological group made the selection of other species of the same biological group in a different region easier. Afterward, the ecotoxicological responses were classified as shown in Table 1. Finally, the data concerning toxicity were plotted according to biological groups and responses.

## 5. The Hazard Concentration (HC_5_) Based on the Species Sensitive Distribution (SSD)

From the EC_50_ data used in the sensitivity profile for biological groups, the species sensitive distribution (SSD) [6] was also generated. When there were two results as the endpoint for the same species, the most sensitive data (lower EC_50_) was used. Finally, the hazard concentration for 5% of the species (HC_5_) was calculated from the SSD according to Posthuma et al. [6] to identify whether the avoidance response can be observed within the concentrations that suppose a risk for the most (5%) sensitive responses.

## 6. Results: Sensitivity Profile by Biological Group

**Figure 1:**
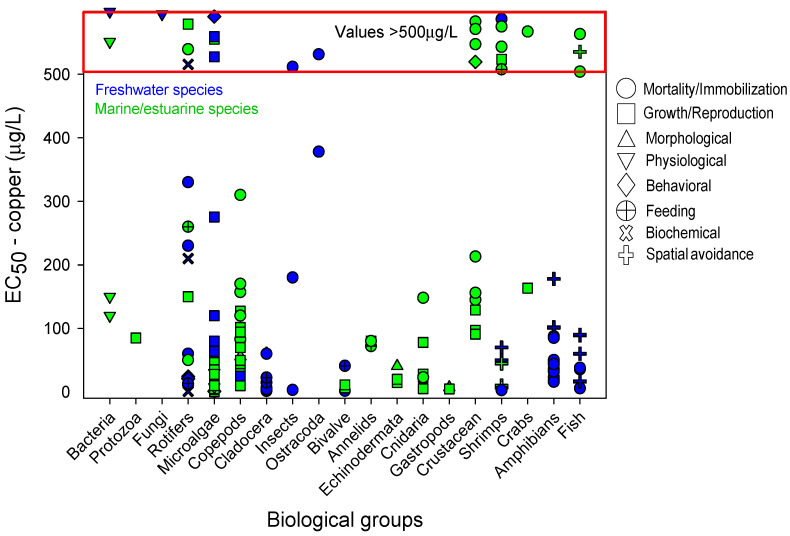
Sensitivity profile for the biological groups exposed to copper-based on the EC_50_ values. The effects, represented by different symbols, were classified according to Table 1. Data in blue and green represent, respectively, freshwater and estuarine/marine species. Data shown in the red zone represent results whose EC_50_ values are higher than 500 µg/L; therefore, the scale should not be considered in this zone (see real data in Table S1).

**Figure 2:**
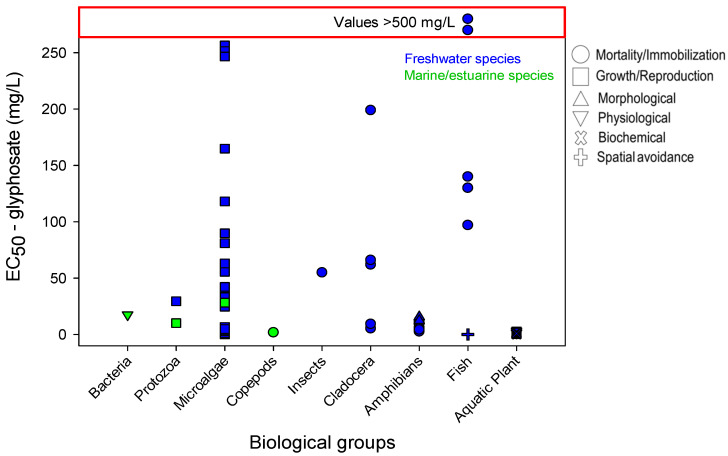
Sensitivity profile for the biological groups exposed to glyphosate-based on the EC_50_ values. The effects, represented by different symbols, were classified according to Table 1. Data in blue and green represent, respectively, freshwater and estuarine/marine species. Data shown in the red zone represent results whose EC_50_ values are higher than 500 mg/L; therefore, the scale should not be considered in this zone (see real data in Table S2).

**Figure 3:**
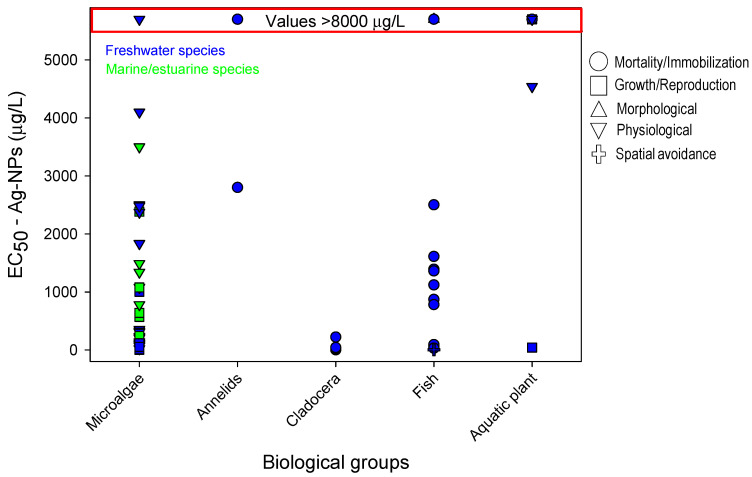
Sensitivity profile for biological groups exposed to Ag-NPs based on the EC_50_ values. The effects, represented by different symbols, were classified according to Table 1. Data in blue and green represent, respectively, freshwater and estuarine/marine species. Data shown in the red zone represent results whose EC_50_ values are higher than 8000 mg/L; therefore, the scale should not be considered in this zone (see real data in Table S3).

As copper is one of the most widely used contaminants in ecotoxicology, the sensitivity profile was more diverse regarding biological groups and endpoints compared to the SPBG of glyphosate and Ag-NPs. In total, 19 biological groups were included for the SPBG of copper (Figure 1), while 9 biological groups were used for glyphosate (Figure 2) and only data for 5 biological groups concerning Ag-NPs were found (Figure 3). Similarly, the responses selected were more diverse for copper (8 groups of response), followed by glyphosate (6 groups of response) and Ag-NPs (5 groups of response). All the data described in the next sections, and their respective references, may be verified in Tables S1–S3. Any reference to the most or least sensitive organism or response discussed in the next sections should be viewed with caution due to intrinsic differences regarding the environmental conditions of the experiments (see more details in the “Avoidance response: relevance and final remarks” section). In addition, it should be taken into consideration that the database used in the current review has its limitation regarding the number of manuscripts revised.

### 6.1. Copper

From the bibliographic review, 160 results were selected for the SPBG to copper (Figure 1; see the complete database in Table S1), 79 for freshwater species, and 81 for marine/estuarine species. The most frequent response was immobility/mortality, which was selected for 13 out of 19 biological groups. The most sensitive biological group for considering the immobility/mortality response was the cladocerans *Bosmina longirostris* with EC_50_ values of 1.4 µg/L.

Growth/reproduction inhibition was the second most common response and was observed in 12 out of 19 biological groups. For this endpoint, microalgae and copepods were proven to be the most sensitive groups; however, microalgae presented the greatest number of sensitive species with EC_50_ values lower than 10 µg/L: e.g., *Isochrysis* aff. *galbana* clone T-ISO (EC_50_ of 0.4 µg/L), *Cylindrotheca closterium* (EC_50_ of 4.7 µg/L), *Selenastrum capricornutum* (EC_50_ of 6 µg/L) *Chlorella* sp. (EC_50_ of 6 µg/L) *Phaeodactylum tricornutum* (EC_50_ of 9 µg/L), *Chlorella autotrophica* (EC_50_ of 9.6 µg/L).

The gastropods *Nassarius dorsatus* (EC_50_ of 4.7 µg/L) and *Haliotis rubra* (EC_50_ of 7.1 µg/L) presented a high sensitivity to copper when growth/reproduction and morphological alterations were considered as the endpoints, respectively. The sensitivity of the rotifers to copper was represented by many different responses, but with ample variation regarding the sensitivity. Although the responses related to biochemical changes, feeding, behavior, and mortality/immobilization were relatively sensitive, the data of the sensitivity profile presented a great dispersion regarding the EC_50_ values. Some species of cnidarian and bivalves seem to be highly sensitive to copper (EC_50_ lower than 10 µg/L; see detail in Table S1).

Regarding avoidance, results were obtained for cladocerans, shrimps, amphibians, and fish. In general, the sensitivity of the organisms to avoiding copper is comparable to the most sensitive values observed in the other biological groups (Figure 1). The most sensitive values for avoidance response were observed for the estuarine/marine shrimp *Palaemon varians* (AC_50_ of 10 µg/L) and *Litopenaeus vannamei* (AC_50_ of 11 µg/L), followed by the freshwater fish *Danio rerio* and *Poecilia reticulata*, both with an AC_50_ of 16 µg/L. The cladoceran *Daphnia longispina* also proved to be able to avoid copper but at a higher concentration (AC_50_ of 65 µg/L). Finally, the amphibians were the groups tested with higher AC_50_ values: *Lithobates catesbeianus* (101 µg/L), *Leptodactylus latrans* (102 µg/L), *Pelophylax perezi* (178 µg/L).

### 6.2. Glyphosate

This sensitivity profile to glyphosate was formed by 105 results from 9 biological groups, the majority (97 results) were from freshwater; only 8 results were used for estuarine/marine species (Figure 2). Although the concentrations at which organisms respond to glyphosate are much higher than those for copper, a similarity was observed regarding the most common endpoints tested: growth/reproduction inhibition and mortality/immobilization.

Microalgae was the most common group included as test organisms, and the data collected (exclusively for growth inhibition) showed high variability in the sensitivity. High variation in the data has also been documented for fish, even considering the same response like mortality/immobilization. On the other hand, the results found for amphibians are very consistent and sensitive; the lowest EC_50_ value observed was for the mortality/immobilization response of *Rana clamitans* (2.7 mg/L). Similarly, the macrophyte *Lemna minor* was shown to be a very sensitive organism. Data of different responses have been found for this species (Table S2) and EC_50_ values as low as 0.09, 0.40, and 1.32 mg/L have been observed for biochemical effects, growth/reproduction, and physiological changes, respectively.

Considerations about the avoidance response must be made with caution because there is only one item of data for exposure to glyphosate. The avoidance shown by *Danio rerio* was observed at very low concentrations (AC_50_ of 0.0015 mg/L), much lower than the second most sensitive response for fish: mortality of *Pimpehales promelas* with EC_50_ of 97 mg/L. The complete database is described in Table S2.

### 6.3. Silver Nanoparticles

Few studies were found for this contaminant. Only five biological groups and five groups of respondents were represented (Figure 3). As observed in the previous sensitivity profile, microalgae presented the highest quantity of data and high variability in the results. In this group, two endpoints were recorded: physiological changes and growth/reproduction. The microalgae species that were shown to be highly sensitive (EC_50_ inferior a 20 µg/L) to Ag-NPs were: *Pseudokirchneriella subcapitata* (EC_50_ of 3.02 µg/L) and *Chlamydomonas reinhardtii* (EC_50_ of 19.8 µg/L). Data from cladocerans also showed a great sensitivity regarding the mortality/immobilization response. The most sensitive response was observed in *Daphnia magna* with an EC_50_ of 0.75 µg/L, followed by *Ceriodaphnia dubia* with an EC_50_ of 5 µg/L.

Regarding fish, the sensitivity to Ag-NPs seems not to be very high. An EC_50_ value as high as 12,600 µg/L was observed for morphological changes in *Oreochromis mossambicus*. On the other hand, the spatial avoidance measured in *D*. *rerio* with an AC_50_ of 2.5 µg/L (data from the current study) was the most sensitive response found for this biological group, and one of the most sensitive when compared to the other biological groups. Growth in aquatic plants did not show great sensitivity to Ag-NPs except for two results (see details of the EC_50_ in Table S3). The annelids seem to be the organisms with the lowest sensitivity to Ag-NP exposure.

## 7. Sensitivity of Avoidance Response According to SSD and HC_5_

**Figure 4:**
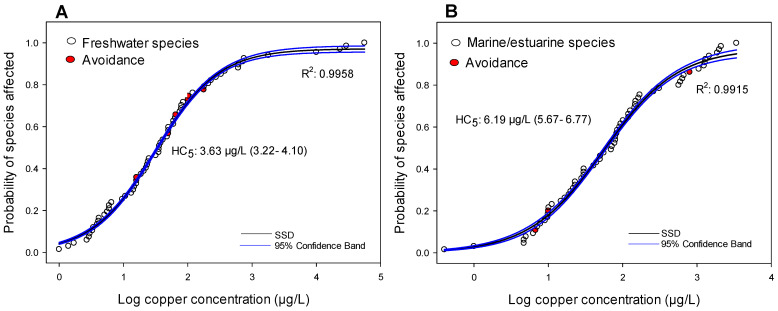
Species sensitive distribution (SSD), and 95% confidence intervals, expressed as the probability of the species being affected according to the log of the copper concentrations, considering the freshwater (**A**) and estuarine/marine (**B**) species. The spatial avoidance response is represented by red circles. Values of hazard concentrations for 5% of the species (HC_5_) and the confidence intervals are also shown for each group of data.

**Figure 5:**
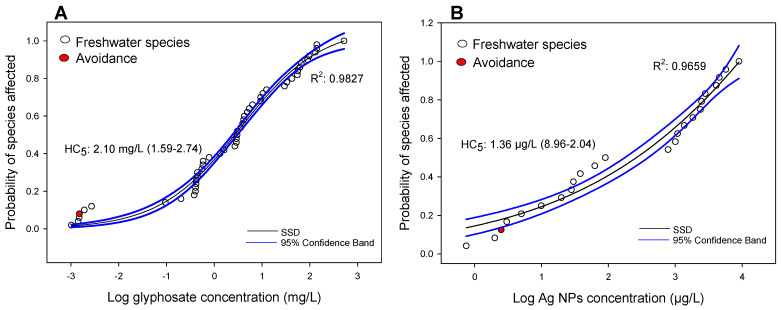
Species sensitive distribution (SSD), and 95% confidence intervals, expressed as the probability of the species being affected according to the log of the glyphosate (**A**) and Ag-NPs (**B**) concentrations, considering the freshwater species. The spatial avoidance response is represented by red circles. Values of hazard concentrations for 5% of the species (HC_5_) and the confidence intervals are also shown for each group of data.

Due to the quantity of data collected for copper, the SSD was divided into two groups: freshwater and estuarine/marine organisms (Figure 4A,B, respectively). For glyphosate and Ag-NPs, only data from freshwater species were used, because very little data for estuarine/marine species were found (Figure 5A,B, respectively). For the four SSDs, the sigmoidal model was statistically significant (*p* < 0.001), and the coefficients of determination were always higher than 0.9 (Figure 4 and Figure 5). Regarding the HC_5_, the values calculated (and confidence intervals) were: 3.63 µg/L (3.22–4.10 µg/L) and 6.19 µg/L (5.67–6.77 µg/L) for freshwater and estuarine/marine species exposed to copper, respectively (Figure 4A,B), and 2.10 mg/L (1.59–2.74 µg/L) and 1.36 µg/L (8.96–2.04 µg/L) for freshwater species exposed to glyphosate and Ag-NPs, respectively (Figure 5A,B).

Considering the SSD curves, the avoidance response (red circles) of the freshwater species exposed to copper seems to be only moderately sensitive compared to other responses. The most sensitive avoidance response was observed at 16 µg/L (*D*. *rerio* and *P*. *reticulata*), which is higher than the HC_5_ value (3.63 µg/L). For estuarine/marine species exposed to copper (Figure 4B), the avoidance responses were distributed at the extremes of the range of sensitivity, where some organisms seem to respond by avoiding low copper concentrations (*Palaemon varians* at 10 µg/L and *Litopenaeus vannamei* at 11 µg/L), while the fish *Rachycentron canadum* (AC_50_ of 800 µg/L) seems to be less responsive. The repellence of copper for the most responsive species (*P*. *varians* and *L*. *vannamei*) occurred at concentrations similar to those affecting the most sensitive species and close to the HC_5_ value (6.19 µg/L).

Analyzing the SSD models for glyphosate and Ag-NPs (Figure 5A,B), the avoidance response appeared as one of the most sensitive endpoints. In the case of glyphosate, the AC_50_ for the fish *D*. *rerio* (0.0015 mg/L) is even lower than the HC_5_ calculated (2.10 mg/L). For Ag-NPs, the AC_50_ for *D*. *rerio* (2.5 µg/L) was slightly higher than the HC_5_ (1.36 µg/L).

## 8. Avoidance Response: Relevance and Final Remarks

This review is an attempt to situate the avoidance response (using the non-forced multi-compartmented exposure approach) into the sensitivity profile by biological groups to assess how sensitive it is. The data of the three contaminants (copper, glyphosate, and Ag-NPs) assessed here showed that avoidance may be considered a very sensitive response, even when compared with the most traditional endpoints such as growth/reproduction inhibition, physiological changes, feeding, mortality/immobilization, and others. As the use of avoidance in multi-compartmented exposure systems, first proposed by Lopes et al. [14], supposes a shift in the paradigm of how organisms are exposed to contaminants and which kind of response is measured (not toxicity, but repellence instead), this approach provides a complementary perspective concerning the risk that contamination may represent to ecosystems.

This response can be applied in different approaches, either by integrating the loss of population due to avoidance with mortality and reproduction [34,35] thereby assessing the decline of populations due to these three responses; simulating changes in the avoidance response and spatial distribution of organisms under scenarios of global changes [36] to predict the loss of populations due to the inhospitable environmental conditions or integrating it with other responses to assess how avoidance can be impaired if some toxic effects occur in organisms [37,38]. As a complementary tool to evaluate how contamination affects the spatial distribution of species, this approach allows researchers to apply a more ecological view of ecotoxicology by simulating some real scenarios, in which the chemical heterogeneity can generate attractive and repellent areas. This spatially broader perspective of the impact of contamination leads to new ecological concepts that can be included in ecotoxicological studies when the avoidance response is used: for instance, colonization [33,39]. This involves assessing the possibility of an area being colonized depending on the levels of contamination that make it attractive. Further, the concept of habitat connectivity/selection [15,16], which considers ecosystems as a spatially continuous landscape throughout which organisms can move. Another new aspect for consideration is habitat fragmentation [40], which integrates the concept of a chemical barrier that prevents the free displacement of species between areas separated by high levels of contamination. Taking into account that the loss of connectivity of habitats is a serious threat to biodiversity, the role of contamination in the chemical fragmentation of habitats should be considered by ecotoxicologists.

Although there is very little data on the avoidance response of organisms to contaminants, this review has shown the high sensitivity of this endpoint. The importance of avoidance as an ecotoxicological endpoint may be deduced from the sensitivity profiles and SSD for copper, glyphosate, and Ag-NPs. In order to protect the environment, it is not only important to know the toxic effect that contaminants produce, but also to what extent their repellence triggers the fleeing of organisms to more favorable areas. We encourage the use of the avoidance response employing non-forced exposure scenarios to make the integration of this response in SSD models more robust, reducing the uncertainties [41] and potentially providing environmentally more protective HC_5_ values [42].

Finally, it is important to consider that, despite the high sensitivity of the avoidance response in non-forced exposure systems, any comparison of the sensitivities of organisms to contaminants should be viewed with great caution. This is necessary because the way organisms respond to chemicals not only depends on the species, their life stages used in the tests, and their origins; but also on several factors such as the environmental conditions under which the organisms are cultured and the tests performed (e.g., the chemical composition of the culture medium, levels of dissolved oxygen, pH, temperature, salinity) [43,44,45].

## Figures and Tables

**Table 1:** Classification of the ecotoxicological responses used in the sensitivity profile of the three chemicals used in the current study by biological group.

Classification of the Effect	Ecotoxicological Responses
Mortality/Immobilization	Death; immobilization
Biochemical	Biomarkers of exposure and effects, enzymes, proteins
Physiological	Respiration rate; heartbeat
Feeding	Ingestion; excretion; post-exposure feeding
Growth/Reproduction	Increase in the body size; population growth (cell numbers)
Morphological	Any morphological alterations
Behavioral	Changes in the movement patterns; all the effects related to swimming; fleeing from a predator; sinking; burrowing
Spatial avoidance	Avoidance behavior related to the habitat selection response measured exclusively in multi-compartmented exposure systems

## Data Availability

Not applicable.

## Supplementary Materials

The following are available online at https://www.mdpi.com/article/10.3390/toxics9110301/s1, Table S1: copper, Table S2: glyphosate, Table S3: Ag-NPs.
