# Supplementary material for: Could Contamination Avoidance Be an Endpoint That Protects the Environment? An Overview on How Species Respond to Copper, Glyphosate, and Silver Nanoparticles
Source: Toxics. 2021 Nov 11;9(11):301. doi: 10.3390/toxics9110301 (PMC8623862; doi:10.3390/toxics9110301)
Supplement: Supplementary file 1 [file toxics-09-00301-s001.zip › toxics-1436094-supplementary.pdf]

toxics

MDPI

Could contamination avoidance be an endpoint that protects the environment? An overview on how species respond to copper, glyphosate and silver nanoparticles

M. Antonella Alcívar, Marta Sendra, Daniel C.V.R. Silva, Enrique González-Ortegón, Julián Blasco, Ignacio Moreno-Garrido and Cristiano V.M. Araújo

Table S1. copper.

Group	Species	Responses /Endpoint	Classification of the ecotoxicological responses	EC_{50} (μg/L)	References
**Freshwater species**					
Bacteria	*Pseudomonas fluorescens*	Respirometry	Physiological	56,100	Pérez-García et al., 1993
Fungi	*Saccharomyces cerevisiae*	Respirometry	Physiological	29,300	Pérez-García et al., 1993
Rotifers	*Brachionus calyciflorus*	Swimming alterations: Speed	Behavioral	24.2	Charoy and Janssen, 1999
		Swimming alterations: Periods of swimming	Behavioral	20.8	Charoy and Janssen, 1999
		Filtration rate	Feeding	10.75	Ferrando and Andreu, 1993
		Ingestion rate	Feeding	13.25	Ferrando and Andreu, 1993
	Lecane hamata	Esterases-inhibition	Biochemical	210	Pérez-Legaspi et al., 2002
		Mortality	Mortality/Immobilization	230	Pérez-Legaspi et al., 2002
	Lecane luna	Esterases-inhibition	Biochemical	620	Pérez-Legaspi et al., 2002
		Mortality	Mortality/Immobilization	60	Pérez-Legaspi et al., 2002
	Lecane quadridentata	Esterases-inhibition	Biochemical	1	Pérez-Legaspi et al., 2002
		Mortality	Mortality/Immobilization	330	Pérez-Legaspi et al., 2002
Microalgae	*Euglena gracilis*	Motility	Behavioral	23,400	Ahmed and Häder, 2010
		Growth inhibition	Growth/Reproduction	1800	Girling et al., 2000
	Selenastrum capricornutum	Cel division rate inhibition	Growth/Reproduction	6	Franklin et al., 2001
	Chlorella sp.	Cel division rate inhibition	Growth/Reproduction	6	Franklin et al., 2001
	Chlamydomonas reinhardi	Growth inhibition	Growth/Reproduction	79	Girling et al., 2000
	Scenedesmus subspicatus	Growth inhibition	Growth/Reproduction	120	Girling et al, 2000
	Scenedesmus quadricauda	Decrease in the surface area	Growth/Reproduction	275.5	Fawaz et al., 2019
		Cell density	Growth/Reproduction	14.29	Fawaz et al., 2019
	Ankistrodesmus angustus	Decrease in the surface area	Growth/Reproduction	75.65	Fawaz et al., 2019
		Cell density	Growth/Reproduction	720.3	Fawaz et al., 2019
	Oscillatoria prolifera	Decrease in the surface area	Growth/Reproduction	50.17	Fawaz et al., 2019
		Cell density	Growth/Reproduction	50.17	Fawaz et al., 2019
	Aphanizomenon gracile	Population growth	Growth/Reproduction	64	Lüderitz and Nicklish, 1989
	Oscillatoria redekei	Population growth	Growth/Reproduction	80	Lüderitz and Nicklish, 1989
	Chlorella autotrophyca	Population growth	Growth/Reproduction	9,6	Moreno-Garrido et al., 2000
		Population growth	Growth/Reproduction	19,3	Moreno-Garrido et al., 2000

		Population growth	Growth/Reproduction	38,3	Moreno-Garrido et al., 2000
Copepods	*Mesocyclops pehpeiensis*	Inhibition	Growth/Reproduction	25	Wong and Pak, 2004
Cladocera	*Bosmina longirostris*	Motility	Mortality/Immobilization	1.4	Koivisto et al., 1992
	Ceriodaphnia cornuta	Motility	Mortality/Immobilization	2.92	Bui et al., 2016
	Ceriodaphnia dubia	Immobilization	Mortality/Immobilization	4.16	Harmon et al., 2003
	Chydorus sphaericus	Motility	Mortality/Immobilization	3.3	Koivisto et al., 1992
	Daphnia ambigua	Immobilization	Mortality/Immobilization	6.53	Harmon et al., 2003
	Daphnia galeata	Motility	Mortality/Immobilization	4.1	Koivisto et al., 1992
	Daphnia longispina	Avoidance	Spatial avoidance	65	Lopes et al., 2004
		Motility	Mortality/Immobilization	60	Lopes et al., 2004
	Daphnia lumholtzi	Motility	Mortality/Immobilization	3.92	Bui et al., 2016
	Daphnia magna	Motility	Mortality/Immobilization	5.1	De Schamphelaere y Janssen, 2002
		Filtration rate	Feeding	14.75	Ferrando and Andreu, 1993
		Ingestion rate	Feeding	22.5	Ferrando and Andreu, 1993
	Daphnia pulex	Motility	Mortality/Immobilization	3.4	Koivisto et al., 1992
Insects	*Adenophlebia auriculata*	Survival	Mortality/Immobilization	180	Gerhardt and Palmer, 1998
	Drunella grandis	Survival	Mortality/Immobilization	3.0	Clements et al., 2013
	Tramea cophysa	Mortality	Mortality/Immobilization	611	Dos Santos Lima et al., 2019
Ostracoda	*Chlamydotheca* sp.	Mortality	Mortality/Immobilization	378	Dos Santos Lima et al., 2019
	Strandesia trispinosa	Mortality	Mortality/Immobilization	750	Dos Santos Lima et al., 2019
Bivalve	*Dreissena polymorpha*	Filtration rate	Feeding	41	Kraak et al., 1994
	Echyridella menziesii	Survival	Mortality/Immobilization	1.7	Clearwater et al., 2013
Shrimps	*Atyaephyra desmarestii*	Avoidance	Spatial avoidance	70	Vera-Vera et al., 2019
		Avoidance	Spatial avoidance	50	Araújo et al., 2019
	Macrobrachium amazonicum	Survival	Mortality/Immobilization	10,010	Soares et al., 2017
	Macrobrachium pantanalense	Survival	Mortality/Immobilization	2.7	Soares et al., 2017
Amphibians	*Ambystoma opacum*	Mortality	Mortality/Immobilization	18.76	Weir et al., 2019.
	Ambystoma talpoideum	Mortality	Mortality/Immobilization	47.88	Weir et al., 2019.
	Ambystoma tigrinum	Mortality	Mortality/Immobilization	35.3	Weir et al., 2019.
	Bufo arenarum	Mortality	Mortality/Immobilization	85	Herkovits y Helguero, 1998.
	Epidalea calamita	Larval mortality	Mortality/Immobilization	87.60	García-Muñoz et al., 2009
		Larval mortality	Mortality/Immobilization	31.85	García-Muñoz et al., 2009
		Larval mortality	Mortality/Immobilization	43.80	García-Muñoz et al., 2009
	Lithobates catesbeianus	Avoidance	Spatial avoidance	101	Araújo et al., 2014
	Leptodactylus latrans	Avoidance	Spatial avoidance	102	Araújo et al., 2014
	Pelophylax perezi	Avoidance	Spatial avoidance	178	Araújo et al., 2014
	Rhinella granulosa	Mortality	Mortality/Immobilization	23.48	Franco-de-Sá and Val, 2014
	Scinax ruber	Mortality	Mortality/Immobilization	36.37	Franco-de-Sá and Val, 2014
		Mortality	Mortality/Immobilization	50.02	Franco-de-Sá and Val, 2014
		Mortality	Mortality/Immobilization	15.9	Franco-de-Sá and Val, 2014
Fish	*Danio rerio*	Feeding Inhibition	Feeding	36	Abdel-moneim et al, 2015
		Avoidance	Spatial avoidance	17	Silva et al., 2018
		Avoidance	Spatial avoidance	60	Araújo et al., 2018
		Avoidance	Spatial avoidance	89	Araújo et al., 2018
		Avoidance	Spatial avoidance	90	Islam et al., 2019
		Avoidance	Spatial avoidance	60	Araújo et al., 2019
		Avoidance	Spatial avoidance	16	Moreira-Santos et al., 2008
	Rasbora sumatrana	Mortality	Mortality/Immobilization	5.6	Shuhaimi-Othman et al., 2015

	Oncorhynchus mykiss	Mortality	Mortality/Immobilization	35.5	Naddy et al., 2015
	Poecilia reticulata	Mortality	Mortality/Immobilization	37.9	Shuhaimi-Othman et al., 2010
		Avoidance	Spatial avoidance	16	Silva et., 2018
**Marine/estuarine species**					
Bacteria	*Vibrio fisherii* (Microtox)	Atenuation of light emission	Physiological	150	Moreno-Garrido et al., 1999
		Atenuation of light emission	Physiological	120	Moreno-Garrido et al., 1999
		Luminescence	Physiological	1300	Toussaint et al., 1995
Protozoa	*Pyrocystis lunula*	Growth rate inhibition	Growth/Reproduction	85	Stauber et al., 2008
Rotifers	*Brachionus plicatilis*	Mortality	Mortality/Immobilization	50	Rotini et al., 2018
	Proales similis	Mortality	Mortality/Immobilization	1060	Snell et al., 2019
		Reproduction	Growth/Reproduction	150	Snell et al., 2019
		Ingestion	Feeding	260	Snell et al., 2019
		Hatching	Growth/Reproduction	3400	Snell et al., 2019
Microalgae	*Cylindrotheca closterium*	Population growth	Growth/Reproduction	27.8	Araújo et al., 2010.
		Chlorophyll fluorescence	Growth/Reproduction	4.7	Araújo et al., 2010.
		Esterase activity	Physiological	7.8	Araújo et al., 2010.
		Population growth	Growth/Reproduction	10.1	Araújo et al.,2010.
	Dunaliella tertiolecta	Inhibition of oxygen rate production	Growth/Reproduction	1461	Franklin et al., 2001
	Gonyaulax tamarensis	Immobilisation	Mortality/Immobilization	1	Anderson and Morel, 1978
	Isochrysis aff. galbana Clone T-ISO	Population growth	Growth/Reproduction	0.4	Moreno-Garrido et al., 2000
		Population growth	Growth/Reproduction	3.6	Moreno-Garrido et al., 2000
		Population growth	Growth/Reproduction	4.4	Moreno-Garrido et al., 2000
	Nannochloris atomus	Population growth	Growth/Reproduction	16.7	Moreno-Garrido et al., 2000
		Population growth	Growth/Reproduction	27.3	Moreno-Garrido et al., 2000
		Population growth	Growth/Reproduction	46.2	Moreno-Garrido et al., 2000
	Phaeodactylum tricornutum	Population growth	Growth/Reproduction	9	Franklin et al., 2001
		Population growth	Growth/Reproduction	9.8	Moreno-Garrido et al., 2000
		Population growth	Growth/Reproduction	34.4	Moreno-Garrido et al., 2000
		Population growth	Growth/Reproduction	35	Moreno-Garrido et al., 2000
	Rhodomonas salina	Inhibition of oxygen rate production	Physiological	30	Moreno-Garrido et al., 1999
		Population growth	Growth/Reproduction	30	Moreno-Garrido et al., 1999
Copepods	*Acartia tonsa*	Egg production / reproduction	Growth/Reproduction	9.9	Lauer and Bianchini, 2010
	Tisbe battagliai	Mortality	Mortality/Immobilization	83.1	Diz, et al., 2009
		Mortality	Mortality/Immobilization	157	Diz, et al., 2009
		Fecundity	Growth/Reproduction	30.08	Diz, et al., 2009
		newborn production	Growth/Reproduction	44.5	Diz, et al., 2009
	Tigriopus fulvus	Mortality	Mortality/Immobilization	310	Biandolino et al., 2018
		Moult naupliar reduction	Morphological	55.8	Biandolino et al., 2018
		Development inhibition	Growth/Reproduction	21.7	Biandolino et al., 2018
		Mortality	Mortality/Immobilization	120	Rotini et al., 2018
		Release of the molt	Growth/Reproduction	70	Rotini et al., 2018
	Gladioferens pectinatus	Survival	Mortality/Immobilization	170	Charry et al., 2019
		Larval development ratio	Growth/Reproduction	49.8	Charry et al., 2019
		Realized offspring	Growth/Reproduction	101.5	Charry et al., 2019
		Potential offspring	Growth/Reproduction	127.1	Charry et al., 2019
		Total offspring	Growth/Reproduction	94.3	Charry et al., 2019

Bivalve	*Crassostera virginica*	Embryo-larval development	Growth/Reproduction	11.2	Arnold et al., 2010
	Mytilus galloprovincialis	Embryo-larval development	Growth/Reproduction	6.28	Arnold et al., 2010
Annelids	*Neanthes arenaceodentata*	Feeding rate	Feeding	72	Rosen and Miller, 2011.
	Ficopomatus enigmaticus	Sperm toxicity/Fertilization rate	Growth/Reproduction	80	Oliva et al., 2017
		Mortality	Mortality/Immobilization	80	Rosen y Miller, 2011.
Echinodermata	*Dendraster excentricus*	Embryo-larval development	Growth/Reproduction	18.9	Arnold et al., 2010
	Paracentrotus lividus	Embryonic malformations	Morphological	40.65	Morroni et al., 2018.
		Fertilization rate	Growth/Reproduction	20	Rotini et al., 2018.
	Strongylocentrotus purpuratus	Embryo-larval development	Growth/Reproduction	14.8	Arnold et al., 2010
Cnidaria	*Acropora aspera*	Inhibition in fertilisation	Growth/Reproduction	78	Gissi et al., 2017
	Aiptasia pallida	Inhibited development	Growth/Reproduction	5	Howe et al., 2014
		Mortality	Mortality/Immobilization	23	Howe et al., 2014
	Exaiptasia pallida	Reproduction	Growth/Reproduction	23	Trenfiled et al., 2017
		Mortality	Mortality/Immobilization	148	Trenfiled et al., 2017
	Platygyra daedalea	Inhibition in fertilisation	Growth/Reproduction	28	Gissi et al., 2017
Gastropods	*Haliotis rubra*	Morphological abnormalities	Morphological	7.10	Gorski and Nugegoda, 2006
	Nassarius dorsatus	Growth rate	Growth/Reproduction	4.7	Trenfield et al., 2016
Crustacean	*Artemia franciscana*	Mortality	Mortality/Immobilization	1280	Rotini et al., 2018
		Swimming speed alteration	Behavioral	638.54	Manfra et al., 2016
	Balanus amphitrite	Survivorship	Mortality/Immobilization	145	Qiu et al., 2005
		Molting success	Growth/Reproduction	97	Qiu et al., 2005
		Survivorship	Mortality/Immobilization	156	Qiu et al., 2005
		Molting success	Growth/Reproduction	91	Qiu et al., 2005
		Survivorship	Mortality/Immobilization	213	Qiu et al., 2005
		Molting success	Growth/Reproduction	129	Qiu et al., 2005
	Exosphaeroma gigas	Immobility	Mortality/Immobilization	6960	Giarratano et al., 2007
		Immobility	Mortality/Immobilization	2110	Giarratano et al., 2007
Shrimps	*Litopenaeus vannamei*	Avoidance	Spatial avoidance	11	Redondo-López et al. (*under review*)
	Palaemon varians	Avoidance	Spatial avoidance	10	Araújo et al., 2020.
		Avoidance	Spatial avoidance	43	Redondo-López et al. (*under review*)
	Penaeus monodon	Mortality	Mortality/Immobilization	1246	Chen and Lin, 2001
		Mortality	Mortality/Immobilization	3078	Chen and Lin, 2001
		Reduced length increase	Growth/Reproduction	667	Chen and Lin, 2001
		Reduced weight gain	Feeding	600	Chen and Lin, 2001
Crabs	*Carcinus maenas*	Mortality	Mortality/Immobilization	2000	Nonnote et al., 1993
	Chasmagnathus granulata	Loss of spawning	Growth/Reproduction	163.4	Zapata et al., 2001.
Fish	*Centropomus parallelus*	Mortality	Mortality/Immobilization	1880	Oliveira et al., 2014
	Pomatoschistus microps	Mortality	Mortality/Immobilization	568.1	Vieira et al., 2009
	Rachycentron canadum	Avoidance	Spatial avoidance	800	Araújo et al., 2016

Table S2. glyphosate.

Group	Species	Responses /Endpoint	Classification of the ecotoxicological responses	EC_{50} mg/L	References
**Freshwater species**					
Protozoa	*Tetrahymena pyriformis*	Growth	Growth/Reproduction	29.5	Tsui and Chu, 2003.
Microalgae	*Scenedesmus vacuolatus*	Growth rate	Growth/Reproduction	4.9	Iummato et al., 2019
		Growth	Growth/Reproduction	4.9	Lummato et al., 2019
	Microcystis aeruginosa	Growth	Growth/Reproduction	6.3	Zhang et el., 2018
		Growth	Growth/Reproduction	0.001486	Hernández-García and Martínez-Jerónimo, 2020
		Growth	Growth/Reproduction	6.7	Lipok et al., 2010
		Growth	Growth/Reproduction	251.4	Lipok et al., 2010
		Growth	Growth/Reproduction	81	Zhang et al., 2018
		Growth	Growth/Reproduction	63	Zhang et al., 2018

	Microcystis aeruginosa (nontoxic)	Growth	Growth/Reproduction	0.441	Smedbol et al., 2017
	Microcystis aeruginosa (toxic)	Growth	Growth/Reproduction	0.446	Smedbol et al., 2017
	Microcystis sp.	Growth	Growth/Reproduction	0.406	Smedbol et al., 2017
	Chlorella pyrenoidosa	Growth	Growth/Reproduction	0.396	Anton et al., 1993.
	Selenastrum capricornutum	Growth inhibition	Growth/Reproduction	5.81	Tsui and Chu, 2003
		Growth	Growth/Reproduction	24.7	Tsui and Chu, 2003
		Growth	Growth/Reproduction	41	Tsui and Chu, 2003
		Growth	Growth/Reproduction	3.92	Tsui and Chu, 2003
		Growth	Growth/Reproduction	5.81	Tsui and Chu, 2003
	Scenedesmus obliquus	Growth	Growth/Reproduction	0.422	Smedbol et al., 2017
	Oocystis lacustris	Growth	Growth/Reproduction	0.415	Smedbol et al., 2017
	Chlamydomonas reinhardtii	Growth	Growth/Reproduction	0.551	Smedbol et al., 2017
	Cryptomonas obovata	Growth	Growth/Reproduction	0.584	Smedbol et al., 2017
	Ankistrodesmus falcatus	Growth	Growth/Reproduction	0.649	Smedbol et al., 2017
		Growth	Growth/Reproduction	0.001411	Hernández-García and Martínez-Jerónimo, 2020
	Pseudokirchneriella subcapitata	Growth	Growth/Reproduction	0.001022	Hernández-García and Martínez-Jerónimo, 2020
	Chlorella vulgaris	Growth	Growth/Reproduction	0.001908	Hernández-García and Martínez-Jerónimo, 2020
		Growth	Growth/Reproduction	0.24	Rodriguez-Gil et al., 2017
		Growth	Growth/Reproduction	0.54	Rodriguez-Gil et al., 2017
		Growth	Growth/Reproduction	118.1	Lipok et al., 2010
		Growth	Growth/Reproduction	292	Lipok et al., 2010
	Scenedesmus incrassatulus	Growth	Growth/Reproduction	0.002702	Hernández-García and Martínez-Jerónimo, 2020
	Raphidocelis subcapitata	Growth	Growth/Reproduction	0.201	Rodriguez-Gil et al., 2017
		Growth	Growth/Reproduction	0.69	Rodriguez-Gil et al., 2017
	Oophila sp.	Growth	Growth/Reproduction	1.98	Rodriguez-Gil et al., 2017
		Growth	Growth/Reproduction	1.61	Rodriguez-Gil et al., 2017
	Chlorella pyrenoidosa	Growth	Growth/Reproduction	0.396	Anton et al., 1993
		Growth	Growth/Reproduction	0.38	Anton et al., 1993
		Growth	Growth/Reproduction	1.08	Anton et al., 1993
	Spirulina platensis	Growth	Growth/Reproduction	33.1	Lipok et al., 2010
	Nostoc puntiforme	Growth	Growth/Reproduction	42.3	Lipok et al., 2010
		Growth	Growth/Reproduction	598.4	Lipok et al., 2010
	Anabaena catenula	Growth	Growth/Reproduction	2.9	Lipok et al., 2010
		Growth	Growth/Reproduction	256.5	Lipok et al., 2010
	Synechocystis aquatilis	Growth	Growth/Reproduction	89.8	Lipok et al., 2010
		Growth	Growth/Reproduction	164.9	Lipok et al., 2010
	Leptolyngbya boryana	Growth	Growth/Reproduction	4.1	Lipok et al., 2010
		Growth	Growth/Reproduction	246.6	Lipok et al., 2010
	Chlorella kessleri (tolerant strain)	Growth	Growth/Reproduction	55.62	Romero et al., 2011
Insects	*Chironomus plumosus*	Immobility	Mortality/Immobilization	55	Folmar et al., 1979
Cladocera	*Ceriodaphnia dubia*	Mortality	Mortality/Immobilization	5.39	Tsui and Chu, 2003
	Daphnia magna	Mortality	Mortality/Immobilization	199	Demetrio et al., 2014
		Mortality	Mortality/Immobilization	9.34	Demetrio et al., 2014
		Immobility	Mortality/Immobilization	62	Alberdi et al., 1996
	Daphnia spinulata	Immobility	Mortality/Immobilization	66	Alberdi et al., 1996
Amphibians	*Lymnodynastes dorsalis*	Mortality	Mortality/Immobilization	3	Mann and Bidwell, 1999
		Mortality	Mortality/Immobilization	12	Mann and Bidwell, 1999
	Litoria moorei	Mortality	Mortality/Immobilization	2.9	Mann and Bidwell, 1999

		Mortality	Mortality/Immobilization	10.4	Mann and Bidwell, 1999
	Heleioporus eyrei	Mortality	Mortality/Immobilization	6.3	Mann and Bidwell, 1999
	Crinia insignifera	Mortality	Mortality/Immobilization	3.6	Mann and Bidwell, 1999
	Xenopus laevis	Mortality	Mortality/Immobilization	9.3	Perkins et al., 2009
	Rana temporaria	Mortality	Mortality/Immobilization	11.1	Wagner et al., 2017
		Mortality	Mortality/Immobilization	10.4	Wagner et al., 2017
		Mortality	Mortality/Immobilization	12.2	Wagner et al., 2017
		Teratogenic	Morphological	15.7	Wagner et al., 2017
		Teratogenic	Morphological	12.4	Wagner et al., 2017
	Rana clamitans	Mortality	Mortality/Immobilization	2.7	Wojtaszek et al., 2004
	Rana pipiens	Mortality	Mortality/Immobilization	4.25	Wojtaszek et al., 2004
Fish	*Salmo gairdneri*	Mortality	Mortality/Immobilization	140	Folmar et al., 1979
	Pimpehales promelas	Mortality	Mortality/Immobilization	97	Folmar et al., 1979
	Ictalurus punctatus	Mortality	Mortality/Immobilization	130	Folmar et al., 1979
	Lepomis macrochirus	Mortality	Mortality/Immobilization	140	Folmar et al., 1979
	Cyprinus carpio	Mortality	Mortality/Immobilization	620	Nešković et al., 1996
		Mortality	Mortality/Immobilization	520.77	Ma and Li, 2015
	Danio rerio	Avoidance	Spatial avoidance	0.0015	Mena et al. (*under review*)
Aquatic Plant	*Lemna minor*	Inhibition of growth rate	Growth/Reproduction	0.40222	Sikorski et al., 2019
		Inhibition in field (Iy)	Growth/Reproduction	0.47996	Sikorski et al., 2019
		Fresh mass of new fronds	Growth/Reproduction	0.57629	Sikorski et al., 2019
		Dry mass	Growth/Reproduction	2.72935	Sikorski et al., 2019
		Shikimic acid content	Biochemical	0.10985	Sikorski et al., 2019
		Chlorophyll SPAD	Physiological	1.47199	Sikorski et al., 2019
		Chlorophyll a content	Physiological	1.35876	Sikorski et al., 2019
		Chlorophyll b content	Physiological	1.32496	Sikorski et al., 2019
		Carotenoid content	Biochemical	1.69507	Sikorski et al., 2019
		Max. quantum efficiency (Fv/Fm)	Physiological	3.04538	Sikorski et al., 2019
		SAMDC activity - S-adenosylmethionine decarboxylase	Biochemical	0.09464	Sikorski et al., 2019
		ODC activity - ornithine decarboxylase	Biochemical	1.44833	Sikorski et al., 2019
		LDC activity - lysine decarboxylase	Biochemical	1.46354	Sikorski et al., 2019
		TDC activity - tyrosine decarboxylase	Biochemical	1.62071	Sikorski et al., 2019
		Tyramine content	Biochemical	0.92612	Sikorski et al., 2019
		Putrescine content	Biochemical	0.86528	Sikorski et al., 2019
		Cadaverine content	Biochemical	0.88894	Sikorski et al., 2019
		Spermidine content	Biochemical	0.78078	Sikorski et al., 2019
		Spermine content	Biochemical	0.90077	Sikorski et al., 2019
		Total biogenic amines content	Biochemical	0.8619	Sikorski et al., 2019
		Peroxidase activity	Biochemical	0.57798	Sikorski et al., 2019
		Catalase activity	Biochemical	0.9971	Sikorski et al., 2019
**Marine/estuarine species**					
Bacteria	*Vibrio fischeri*	Luminescence	Physiological	17.5	Tsui and Chu, 2003

Protozoa	*Euplotes vannus*	Growth	Growth/Reproduction	10.1	Tsui and Chu, 2003
Microalgae	*Skeletonema costatum*	Growth	Growth/Reproduction	1.85	Tsui and Chu, 2003
		Growth	Growth/Reproduction	5.89	Tsui and Chu, 2003
		Growth	Growth/Reproduction	3.35	Tsui and Chu, 2003
		Growth	Growth/Reproduction	2.27	Tsui and Chu, 2003
	Arthrospira fusiformis	Growth	Growth/Reproduction	28.2	Lipok et al., 2010
Copepods	*Acartia tonsa*	Mortality	Mortality/Immobilization	1.77	Tsui and Chu, 2003

Table S3. Ag-NPs.

Group	Species	Responses /Endpoint	Classification of the ecotoxicological responses	EC_{50} (ug/L)	References
**Freshwater species**					
Microalgae	*Raphidocelis subcapitata*	Photosynthetic efficiency	Physiological	21,200	Wang et al., 2012
		Photosynthetic efficiency	Physiological	4100	Wang et al., 2012
	Chlamydomonas reinhardtii	Photosynthetic yield	Physiological	20.3	Navarro et al., 2008
		Photosynthetic yield	Physiological	19.8	Navarro et al., 2008
		Photosynthetic yield	Physiological	21.5	Navarro et al., 2008
		Photosynthetic yield	Physiological	356	Navarro et al., 2008
		Photosynthetic yield	Physiological	113	Navarro et al., 2008
		Photosynthetic yield	Physiological	95	Navarro et al., 2008
		Photosynthetic yield	Physiological	86	Navarro et al., 2008
		Photosynthetic yield	Physiological	89	Navarro et al., 2008
		Photosynthetic yield	Physiological	321	Navarro et al., 2012
		Photosynthetic yield	Physiological	139	Navarro et al., 2012
		Photosynthetic yield	Physiological	231	Navarro et al., 2012
		Photosynthetic yield	Physiological	306	Navarro et al., 2012
		Photosynthetic yield	Physiological	84	Navarro et al., 2012
		Photosynthesis	Physiological	138	Piccapietra et al., 2012
		Photosynthesis	Physiological	62.6	Piccapietra et al., 2012
		Photosynthesis	Physiological	1078	Dewez & Oukarroum, 2012
	Thalassiosira weissflogii	Growth	Growth/Reproduction	1003	Bielmyer-Fraser et al., 2014
	Pseudokirchneriella subcapitata	Growth	Growth/Reproduction	3.02	Angel et al., 2013
		Growth	Growth/Reproduction	3.2	Angel et al., 2013
		Growth	Growth/Reproduction	32.4	Ribeiro et al., 2014
		Growth	Growth/Reproduction	9.9	Kennedy et al., 2014
		Growth	Growth/Reproduction	115.4	Tuominen et al., 2013
		Growth	Growth/Reproduction	51.8	Tuominen et al., 2013
	Synechococcus sp.	Growth	Growth/Reproduction	1079	Burchardt et al., 2012
	Mixed periphyton	PS yield	Physiological	8953	Gil - Allué et el., 2015
		respiration	Physiological	2373	Gil - Allué et el., 2015
		GLU activity	Physiological	1834	Gil - Allué et el., 2015
		LAP activity	Physiological	2480	Gil - Allué et el., 2015
Annelids	*Caenorhabditis elegans*	Mortality	Mortality/Immobilization	13400	Ellegaard-Jensen et al., 2012
		Mortality	Mortality/Immobilization	2800	Ellegaard-Jensen et al., 2012
Cladocera	*Daphnia magna*	Mortality	Mortality/Immobilization	4	Asghari et al., 2012
		Mortality	Mortality/Immobilization	2	Asghari et al., 2012
		Inmmobilization	Mortality/Immobilization	0.75	Lee et al., 2012
		Immobilization	Mortality/Immobilization	1	Kim et al., 2011
		Immobilization	Mortality/Immobilization	1.4	Kim et al., 2011
		Immobilization	Mortality/Immobilization	7	Hoheisel et al. 2012
		Immobilization	Mortality/Immobilization	10	Hoheisel et al. 2012

		Immobilization	Mortality/Immobilization	20	Hoheisel et al. 2012
		Immobilization	Mortality/Immobilization	30	Hoheisel et al. 2012
		Immobilization	Mortality/Immobilization	30	Hoheisel et al. 2012
		Immobilization	Mortality/Immobilization	10	Jo et al., 2012
		Immobilization	Mortality/Immobilization	10	Poynton et al., 2012
		Immobilization	Mortality/Immobilization	4	Poynton et al., 2012
		Immobilization	Mortality/Immobilization	30	Zhao and Wang, 2011
		Immobilization	Mortality/Immobilization	2	Zhao and Wang, 2011
		Immobilization	Mortality/Immobilization	1	Zhao and Wang, 2011
		Immobilization	Mortality/Immobilization	1	Kim et al., 2011
		Immobilization	Mortality/Immobilization	2	Kim et al., 2011
		Immobilization	Mortality/Immobilization	20	Blinova et al., 2012
		Immobilization	Mortality/Immobilization	40	Blinova et al., 2012
	Ceriodaphnia dubia	mortality and/or immobilization	Mortality/Immobilization	221	McLaughlin and Bonzongo, 2012.
		Immobilization	Mortality/Immobilization	5	Kennedy et al., 2012
		Immobilization	Mortality/Immobilization	30	Kennedy et al., 2012
	Daphnia pulex	Immobilization	Mortality/Immobilization	40	Griffitt et al. 2008
Fish	*Oryzias latipes*	Survival	Mortality/Immobilization	28	Kim et al. 2011
		Survival	Mortality/Immobilization	67	Kim et al. 2011
		Mortality	Mortality/Immobilization	1380	Wu and Zhou, 2013
		Mortality	Mortality/Immobilization	1120	Wu and Zhou, 2013
		Mortality	Mortality/Immobilization	870	Wu and Zhou, 2013
		Mortality	Mortality/Immobilization	10,000	Kwok et al., 2012
		Mortality	Mortality/Immobilization	2500	Kwok et al., 2012
		Mortality	Mortality/Immobilization	10,000	Kwok et al., 2012
		Mortality	Mortality/Immobilization	30	Chae et al., 2009
		Mortality	Mortality/Immobilization	1390	Kashiwada et al., 2012
	Pimephales promelas	Mortality	Mortality/Immobilization	90	Hoheisel et al., 2012.
	Danio rerio	Mortality	Mortality/Immobilization	1610	Wang et al., 2012
		Mortality	Mortality/Immobilization	1360	Wang et al., 2012
		Mortality	Mortality/Immobilization	780	Wang et al., 2012
		Avoidance	Spatial avoidance	2.5	The current study
	Oreochromis mossambicus	Morphological	Morphological	12,600	Govindasamy and Rahuman, 2012
	Pimephales promelas	Mortality	Mortality/Immobilization	9400	Laban et al., 2010

		Mortality	Mortality/Immobilization	11,250	Laban et al., 2010
		Mortality	Mortality/Immobilization	10,600	Laban et al., 2010
		Mortality	Mortality/Immobilization	1360	Laban et al., 2010
Aquatic plant	*Lemna minor*	Frond number	Growth/Reproduction	38.06	Gubbins et al., 2011
		Frond number	Growth/Reproduction	42.51	Gubbins et al., 2011
	Spirodela polyrhiza	Fresh weight	Growth/Reproduction	13,670	Jiang et al., 2012
		Dry weight	Mortality/Immobilization	13,670	Jiang et al., 2012
		ChlA	Physiological	16,100	Jiang et al., 2012
		Phosphate–phosphorus	Physiological	17,330	Jiang et al., 2012
		Nitrate–nitrogen	Physiological	4540	Jiang et al., 2012
**Marine/estuarine species**					
Microalgae	*Phaeodactylum tricornutum*	Growth	Growth/Reproduction	162,5	Sendra et al, 2017
		Growth	Growth/Reproduction	100,3	Pérez et al., 2014
		Growth	Growth/Reproduction	2384	Angel et al., 2013
		Growth	Growth/Reproduction	6925	Angel et al., 2013
	Chlorella autotrophica	Growth	Growth/Reproduction	570	Sendra et al., 2018
		Cell viability	Physiological	320	Sendra et al., 2018
		cell complexity	Physiological	1490	Sendra et al., 2018
		EQY	Physiological	1340	Sendra et al., 2018
		active Chlorophyll	Physiological	220	Sendra et al., 2018
		ROS	Physiological	200	Sendra et al., 2018
	Dunaliella salina	Growth	Growth/Reproduction	640	Sendra et al., 2018
		Chla	Physiological	3500	Sendra et al., 2018
		EQY	Physiological	2500	Sendra et al., 2018
		active Chlorophyll	Physiological	780	Sendra et al., 2018
	Cylindrotheca closterium	Growth	Growth/Reproduction	239.5	Pérez et al., 2014
	Nitzchia palea	Growth	Growth/Reproduction	76.6	Pérez et al., 2014
	Thalassiosira pseudonana	Growth	Growth/Reproduction	1079	Burchardt et al., 2012

References

1. Abdel-Moneim, A.; Moreira-Santos, M.; Ribeiro, R. A short-term sublethal toxicity assay with zebra fish based on preying rate and its integration with mortality. *Chemosphere*. **2015**, *120*, 568–574.
2. Ahmed, H.; Häder, D.P. A fast algal bioassay for assessment of copper toxicity in water using *Euglena gracilis*. *J. Appl. Phycol.* **2010**, *22*(6), 785–792.
3. Anderson, D.M.; Morel, F.M. Copper sensitivity of *Gonyaulax tamarensis 1*. *Limnology and Oceanography*. **1978**, *23*(2), 283–295.
4. Araújo, C.V.; Diz, F.R.; Lubián, L.M.; Blasco, J.; Moreno-Garrido, I. Sensitivity of *Cylindrotheca closterium* to copper: Influence of three test endpoints and two test methods. *Sci. Total Environ.* **2010**, *408*(17), 3696–3703.
5. Araújo, C.V.; Shinn, C.; Moreira-Santos, M.; Lopes, I.; Espíndola, E.L.; Ribeiro, R. Copper-driven avoidance and mortality in temperate and tropical tadpoles. *Aquat. Toxicol.* **2014**, *146*, 70–75.
6. Araújo, C.V.; Cedeño-Macías, L.A.; Vera-Vera, V.C.; Salvatierra, D.; Rodríguez, E.N.; Zambrano, U.; Kuri, S. Predicting the effects of copper on local population decline of 2 marine organisms, cobia fish and whiteleg shrimp, based on avoidance response. *Environ. Toxicol. Chem.* **2016**, *35*(2), 405–410.
7. Araújo, C.V.; Roque, D.; Blasco, J.; Ribeiro, R.; Moreira-Santos, M.; Toribio, A.; Aguirre, E.; Barro, S. Stress-driven emigration in complex field scenarios of habitat disturbance: The heterogeneous multi-habitat assay system (HeMHAS). *Sci Total Environ.* **2018**; *644*, 31–36.
8. Araújo, C.V.; Pontes, J.R.S.; Blasco, J. Might the interspecies interaction between fish and shrimps change the pattern of their avoidance response to contamination? *Ecotoxicol. Environ. Saf.* **2019**, *186*, 109757.
9. Araújo, C.V.; Rodríguez-Romero, A.; Fernández, M.; Sparaventi, E.; Medina, M.M.; Tovar-Sánchez, A. Repellency and mortality effects of sunscreens on the shrimp *Palaemon varians*: Toxicity dependent on exposure method. *Chemosphere*. **2020**, 127190.
10. Arnold, W.R.; Cotsifas, J.S.; Ogle, R.S.; DePalma, S.G.; Smith, D.S. A comparison of the copper sensitivity of six invertebrate species in ambient salt water of varying dissolved organic matter concentrations. *Environ. Toxicol. Chem.* **2010**, *29*(2), 311–319.
11. Biandolino, F.; Parlapiano, I.; Faraponova, O.; Prato, E. Effects of short- and long-term exposures to copper on lethal and reproductive endpoints of the harpacticoid copepod *Tigriopus fulvus*. *Ecotoxicolo. Environ. Saf.* **2018**, *147*, 327–333.
12. Bui, T.K.L.; Do-Hong, L.C.; Dao, T.S.; Hoang, T.C. Copper toxicity and the influence of water quality of Dongnai River and Mekong River waters on copper bioavailability and toxicity to three tropical species. *Chemosphere*. **2016**, *144*, 872–878.

13. Charoy, C.; Janssen, C.R. The swimming behaviour of *Brachionus calyciflorus* (rotifer) under toxic stress: II. Comparative sensitivity of various behavioural criteria. *Chemosphere*. **1999**, *38*(14), 3247–3260.
14. Charry, M.P.; Northcott, G.L.; Gaw, S.; Keesing, V.; Costello, M.J.; Tremblay L. A. Development of acute and chronic toxicity bioassays using the pelagic copepod *Gladioferens pectinatus*. *Ecotoxicol. Environ. Saf.*, **2019**, *174*, 611–617.
15. Chen, J.C.; Lin, C.H. (2001). Toxicity of copper sulfate for survival, growth, molting and feeding of juveniles of the tiger shrimp, *Penaeus monodon*. Aquaculture. **2001**, *192*(1), 55–65.
16. Clearwater, S.J.; Thompson, K.J.; Hickey, C.W. Acute toxicity of copper, zinc, and ammonia to larvae (Glochidia) of a native freshwater mussel *Echyridella menziesii* in New Zealand. *Arch. Environ. Contam. Toxicol.* **2014**, *66*, 213–226.
17. Clements, W.H.; Cadmus, P.; Brinkman, S.F. Responses of aquatic insects to Cu and Zn in stream microcosms: Understanding differences between single species tests and field responses. *Environ. Sci. Technol.* **2003**, *47*(13), 7506–7513.
18. De Schamphelaere, K.A.; Janssen, C.R. A biotic ligand model predicting acute copper toxicity for *Daphnia magna*: The effects of calcium, magnesium, sodium, potassium, and pH. *Environ. Sci. Technol*. **2002**, *36*(1), 48–54.
19. Diz, F.R.; Araújo, C.V.; Moreno-Garrido, I.; Hampel, M.; Blasco, J. Short-term toxicity tests on the harpacticoid copepod *Tisbe battagliai*: Lethal and reproductive endpoints. *Ecotoxicol. Environ. Saf.* **2009**, *72*(7), 1881–1886.
20. Dos Santos Lima, J.C.; Neto, A.J.G.; de Pádua Andrade, D.; Freitas, E.C.; Moreira, R.A.; Miguel, M.; Daam, A.; Rocha, O. Acute toxicity of four metals to three tropical aquatic invertebrates: The dragonfly *Tramea cophysa* and the ostracods *Chlamydotheca* sp. and *Strandesia trispinosa*. *Ecotoxicol. Environ. Saf.* **2019**, *180*, 535–541.
21. Fawaz, E.G.; Kamareddine, L.A.; Salam, D.A. Effect of algal surface area and species interactions in toxicity testing bioassays. *Ecotoxicol. Environ. Saf.* **2019**, *174*, 584–591.
22. Ferrando, M.D.; Andreu, E. Feeding behavior as an index of copper stress in Daphnia magna and *Brachionus calyciflorus. Comp. Biochem. Physiol. C: Pharmacol. Toxicol.*. 1993, 106(2), 327–331.
23. Franco-de-Sá, J.F.O.; Val, A.L. Copper toxicity for *Scinax ruber* and *Rhinella granulosa* (Amphibia: Anura) of the Amazon: Potential of Biotic Ligand Model to predict toxicity in urban streams. *Acta Amazonica*. **2014**, *44*(4), 491–498.
24. Franklin, N.M.; Adams, M.S.; Stauber, J.L.; Lim, R.P. Development of an improved rapid enzyme inhibition bioassay with marine and freshwater microalgae using flow cytometry. *Arch. Environ. Contam. Toxicol*. **2001**, *40*(4), 469–480.
25. Girling, A.E.; Pascoe, D.; Janssen, C.R.; Peither, A.; Wenzel, A.; Schäfer, H., Persoone, G. Development of methods for evaluating toxicity to freshwater ecosystems. *Ecotoxicol. Environ. Saf.* **2000**, *45*(2), 148–176.
26. Gerhardt, A.; Palmer, C. Copper tolerances of *Adenophlebia auriculata* (Eaton) 1884 (Insecta: Ephemeroptera) and *Burnupia stenochorias* Cawston 1932 (Gastropoda: Ancylidae) in indoor artificial streams. *Sci. Total Environ*. **1998**, *215*(3), 217–229.
27. García-Muñoz, E.; Guerrero, F.; Parra, G. Effects of copper sulfate on growth, development, and escape behavior in *Epidalea calamita* embryos and larvae. *Arch. Environ. Contam.Toxicol*. **2009**, *56*(3), 557.
28. Giarratano, E.; Comoglio, L.; Amin, O. Heavy metal toxicity in *Exosphaeroma gigas* (Crustacea, Isopoda) from the coastal zone of Beagle Channel. *Ecotoxicol. Environ. Saf.* **2007**, *68*(3), 451–462.
29. Gissi, F.; Stauber, J.; Reichelt-Brushett, A.; Harrison, P.L.; Jolley, D.F. Inhibition in fertilisation of coral gametes following exposure to nickel and copper. *Ecotoxicol. Environ. Saf.* **2017**, *145*, 32–41.
30. Gorski, J.; Nugegoda, D. Sublethal toxicity of trace metals to larvae of the blacklip abalone, *Haliotis rubra*. *Environ. Toxicol. Chem*. **2006**, *25*(5), 1360–1367.
31. Harmon, S.M.; Specht, W.L.; Chandler, G.T. A comparison of the daphnids *Ceriodaphnia dubia* and *Daphnia ambigua* for their utilization in routine toxicity testing in the southeastern United States. *Arch. Environ. Contam. Toxicol*. **2003**, *45*(1), 0079–0085.
32. Herkovits, J.; Helguero, L.A. Copper toxicity and copper–zinc interactions in amphibian embryos. *Sci. Total Environ*. **1998**, *221*(1), 1–10.
33. Howe, P.L.; Reichelt-Brushett, A.J.; Clark, M.W. Development of a chronic, early life-stage sub-lethal toxicity test and recovery assessment for the tropical zooxanthellate sea anemone *Aiptasia pulchella*. *Ecotoxicol. Environ. Saf.* **2014**, *100*, 138–147.
34. Islam, M.A.; Blasco, J.; Araújo, C.V. Spatial avoidance, inhibition of recolonization and population isolation in zebrafish (*Danio rerio*) caused by copper exposure under a non-forced approach. *Sci. Total Environ*. **2019**, *653*, 504–511.
35. Kraak, M.H.; Toussaint, M.; Lavy, D.; Davids, C. Short-term effects of metals on the filtration rate of the zebra mussel *Dreissena polymorpha*. *Environ. Pollut*. **1994**, *84*(2), 139–143.
36. Koivisto, S.; Ketola, M.; Walls, M. Comparison of five cladoceran species in short-and long-term copper exposure. *Hydrobiologia*. **1992**, *248*(2), 125–136.
37. Laurer, M.M.; Bianchini, A. Chronic copper toxicity in the estuarine copepod *Acartia tonsa* at different salinities. *Environ. Toxicol. Chem.* **2010**, *29*, 2297–2303.
38. Lopes, I.; Baird, D.J.; Ribeiro, R. Avoidance of copper contamination by field populations of *Daphnia longispina*. *Environ. Toxicol. Chem.*. **2004**, *23*(7), 1702–1708.
39. Lüderitz, V.; Nicklisch, A. The effect of pH on copper toxicity to blue-green algae. *Int. Rev. Der Gesamten Hydrobiol. Und Hydrographie.* **1989**. *74*(3), 283–291.
40. Manfra, L.; Canepa, S.; Piazza, V.; Faimali, M. Lethal and sublethal endpoints observed for Artemia exposed to two reference toxicants and an ecotoxicological concern organic compound. *Ecotoxicol. Environ. Saf.* **2016**, *123*, 60–64.
41. Moreira-Santos, M.; Donato, C.; Lopes, I.; Ribeiro, R. Avoidance tests with small fish: Determination of the median avoidance concentration and of the lowest-observed-effect gradient. *Environ. Toxicol. Chem.*. **2008**, *27*(7), 1576–1582.
42. Moreno Garrido, I.; Lubián, L.M.; Soares, A.M.V.M. Oxygen production rate as a test for determining toxicity of copper to *Rhodomonas salina* Hill and Wehterbee (Cryptophyceae). *Bull. Environ. Contam. Toxicol.* **1999**, *62*(6), 776–782.

43. Moreno-Garrido, I.; Lubián, L.M.; Soares, A.M. Influence of cellular density on determination of EC50 in microalgal growth inhibition tests. *Ecotoxicol. Environ. Saf.* **2000**, *47*(2), 112–116.
44. Morroni, L.; Pinsino, A.; Pellegrini, D.; Regoli, F. Reversibility of trace metals effects on sea urchin embryonic development. *Ecotoxicol. Environ. Saf.* **2018**, *148*, 923–929.
45. Nonnotte, L.; Boitel, F.; Truchot, J.P. Waterborne copper causes gill damage and hemolymph hypoxia in the shore crab *Carcinus maenas*. *Canadian* J. Zool. **1993**, *71*(8), 1569–1576.
46. Naddy, R.B.; Cohen, A.S.; Stubblefield, W.A. The interactive toxicity of cadmium, copper, and zinc to *Ceriodaphnia dubia* and rainbow trout (Oncorhynchus mykiss). *Environ. Toxicol. Chem.* **2015**, *34*(4), 809–815.
47. Oliveira, B.L.; Fernandes, L.F.L.; Bianchini, A.; Chiparri-Gomes, A.R.; Silva, B.F.; Brandão, G.P.; Gomes, L.C. Acute copper toxicity in juvenile fat snook *Centropomus parallelus* (Teleostei: Centropomidae) in sea water. *Neotrop. Ichthyol.* **2014**, *12*, 845–852.
48. Rosen, G.; Miller, K. A postexposure feeding assay using the marine polychaete Neanthes arenaceodentata suitable for laboratory and in situ exposures. *Environ. Toxicol. Chem.* **2011**, *30*(3), 730–737.
49. Rotini, A.; Gallo, A.; Parlapiano, I.; Berducci, M.T.; Boni, R.; Tosti, E.; Prato, E.; Maggi, C.; Cicero, A.M.; Migliore, L.; Manfra, L. Insights into the CuO nanoparticle ecotoxicity with suitable marine model species. *Ecotoxicol. Environ. Saf.* **2018**, *147*, 852–860.
50. Pérez-García, A.; Codina, J.C.; Cazorla, F.M.; de Vicente, A. Rapid respirometric toxicity test: Sensitivity to metals. *Bull. Environ. Contam. Toxicol.* **1993**, *50*, 703–708.
51. Pérez-Legaspi, I.A.; Rico-Martínez, R.; Pineda-Rosas, A. Toxicity testing using esterase inhibition as a biomarker in three species of the genus Lecane (Rotifera). *Environ. Toxicol. Chemistry.* **2002**, *21*(4), 776–782.
52. Qiu, J.W.; Thiyagarajan, V.; Cheung, S.; Qian, P.Y. Toxic effects of copper on larval development of the barnacle *Balanus amphitrite*. *Mar. Pollut. Bulletin.* **2005**, *51*(8–12), 688–693.
53. Redondo-López, S.; Mena, F.; González-Ortegón, E.; Araújo, C.V.M. Dissimilar behavioral and spatial avoidance responses by shrimps from tropical and temperate environments exposed to copper. *Environ Toxicol Chem. (under review).*
54. Shuhaimi-Othman, M.; Yakub, N.; Ramle, N.A.; Abas, A. Comparative toxicity of eight metals on freshwater fish. *Toxicol. Indust. Health*. **2015**, *31*(9), 773–782.
55. Silva, D.C.; Araújo, C.V.; Marassi, R.J.; Cardoso-Silva, S.; Neto, M.B.; Silva, G.C.; Ribeiro, R.; Silva, F.T.; Paiva, T.C.B.; Pompêo, M.L. Influence of interspecific interactions on avoidance response to contamination. *Sci. Total Environ.* **2018**, *642*, 824–831.
56. Soares, M.P.; Jesus, F.; Almeida, A.R.; Zlabek, V.; Grabic, R.; Domingues, I.; Hayd, L. Endemic shrimp *Macrobrachium pantanalense* as a test species to assess potential contamination by pesticides in Pantanal (Brazil). *Chemosphere*. **2017**, *168*, 1082–1092.
57. Snell, T.W.; Johnston, R.K.; Matthews, A.B.; Park, N.; Berry, S.; Brashear, J. Using *Proales similis* (Rotifera) for toxicity assessment in marine waters. *Environ. Toxicol.* **2019**, *34*(5), 634–644.
58. Stauber, J.L.; Binet, M.T.; Bao, V.W.; Boge, J.; Zhang, A.Q.; Leung, K.M.; Adams, M.S. Comparison of the Qwiklite™ algal bioluminescence test with marine algal growth rate inhibition bioassays. *Environ. Toxicol.* **2008**. *23*(5), 617–625.
59. Trenfield, M.A.; van Dam, J.W.; Harford, A.J.; Parry, D.; Streten, C.; Gibb, K.; van Dam, R.A. A chronic toxicity test for the tropical marine snail *Nassarius dorsatus* to assess the toxicity of copper, aluminium, gallium, and molybdenum. *Environ. Toxicol. Chem*. **2016**, *35*(7), 1788–1795.
60. Trenfield, M.A.; van Dam, J.W.; Harford, A.J.; Parry, D.; Streten, C.; Gibb, K.; van Dam, R.A. Assessing the chronic toxicity of copper and aluminium to the tropical sea anemone *Exaiptasia pallida*. *Ecotoxicol. Environ. Saf.* **2017**, *139*, 408–415.
61. Toussaint, M.W.; Shedd, T.R.; van der Schalie, W.H.; Leather, G.R. A comparison of standard acute toxicity tests with rapid-screening toxicity tests. *Environ. Toxicol. Chem*. **1995**, *14*(5), 907–915.
62. Vera-Vera, V.C.; Guerrero, F.; Blasco, J.; Araújo, C.V. Habitat selection response of the freshwater shrimp *Atyaephyra desmarestii* experimentally exposed to heterogeneous copper contamination scenarios. *Sci. Total Environ*. **2019**, *662*, 816–823.
63. Vieira, L.R.; Gravato, C.; Soares, A.M.V.M.; Morgado, F.; Guilhermino, L. Acute effects of copper and mercury on the estuarine fish *Pomatoschistus microps*: Linking biomarkers to behaviour. *Chemosphere*. **2009**, *76*(10), 1416–1427.
64. Weir, S.M.; Yu, S.; Scott, D.E.; Lance, S.L. Acute toxicity of copper to the larval stage of three species of ambystomatid salamanders. *Ecotoxicology*. **2019**, *28*(9), 1023–1031.
65. Wong, C.K.; Pak, A.P. Acute and subchronic toxicity of the heavy metals copper, chromium, nickel, and zinc, individually and in mixture, to the freshwater copepod *Mesocyclops pehpeiensis*. *Bull. Environ. Contam. Toxicol.* **2004**, 73, 190–196.
66. Zapata, V.; Greco, L.L.; Rodríguez, E.M. Effect of copper on hatching and development of larvae of the estuarine crab *Chasmagnathus granulata* (Decapoda, Brachyura). *Environ. Toxicol. Chem*. **2001**, *20*(7), 1579–1583.
67. Alberdi, J.L.; Sáenz, M.E.; Di Marzio, W.D.; Tortorelli, M.C. Comparative acute toxicity of two herbicides, paraquat and glyphosate, to *Daphnia magna* and *D. spinulata*. *Bull. Environ. Contam. Toxicol.* **1996**, *57*(2), 229–235.
68. Anton, F.A.; Ariz, M.; Alia, M. Ecotoxic effects of four herbicides (glyphosate, alachlor, chlortoluron and isoproturon) on the algae *Chlorella pyrenoidosa* Chick. *Sci. Total Environ*. **1993**, *134*, 845–851.
69. Demetrio, P.M.; Bonetto, C.; Ronco, A.E. The effect of cypermethrin, chlorpyrifos, and glyphosate active ingredients and formulations on *Daphnia magna* (Straus). *Bull. Environ. Contam. Toxicol.* **2014**, *93*(3), 268–273.
70. Folmar, L.C.; Sanders, H.O.; Julin, A.M. Toxicity of the herbicide glyphosate and several of its formulations to fish and aquatic invertebrates. *Arch. Environ. Contam. Toxicol*. **1979**, *8*(3), 269–278.
71. Hernández-García, C.I.; Martínez-Jerónimo, F. Multistressor negative effects on an experimental phytoplankton community. The case of glyphosate and one toxigenic cyanobacterium on *Chlorophycean microalgae*. *Sci. Total Environ*. **2020**, *717*, 137186.
72. Lipok, J.; Studnik, H.; Gruyaert, S. The toxicity of Roundup® 360 SL formulation and its main constituents: Glyphosate and isopropylamine towards non-target water photoautotrophs. *Ecotoxicol. Environ. Saf.* **2010**, *73*(7), 1681–1688.

73. Ma, J.; Li, X. Alteration in the cytokine levels and histopathological damage in common carp induced by glyphosate. *Chemosphere*. **2015**, *128*, 293–298.
74. Mann, R.M.; Bidwell, J.R. The toxicity of glyphosate and several glyphosate formulations to four species of southwestern Australian frogs. *Arch. Environ. Contamin. Toxicology*. **1999**, *36*(2), 193–199.
75. Mena, F.; Romero, A.; Blasco, J.; Araújo, C.V.M. Can a mixture of agrochemicals (glyphosate, chlorpyrifos and chorothalonil) mask the perception of an individual chemical? A hidden trap underlying ecological risk. *Environ. Pollut. (under review)*.
76. Nešković, N.K.; Poleksić, V.; Elezović, I.; Karan, V.; Budimir, M. Biochemical and histopathological effects of glyphosate on carp, *Cyprinus carpio L. Bull. Environ. Contam. Toxicol.* **1996**, *56*(2), 295–302.
77. Perkins, P.J.; Boermans, H.J.; Stephenson, G.R. Toxicity of glyphosate and triclopyr using the frog embryo teratogenesis assay—Xenopus. *Environ. Toxicol. Chem.: An International Journal*. **2000**, *19*(4), 940–945.
78. Rodriguez-Gil, J.L.; Prosser, R.; Poirier, D.; Lissemore, L.; Thompson, D.; Hanson, M.; Solomon, K.R. Aquatic hazard assessment of MON 0818, a commercial mixture of alkylamine ethoxylates commonly used in glyphosate-containing herbicide formulations. Part 1: Species sensitivity distribution from laboratory acute exposures. *Environ. Toxicol. Chem*. **2017**, *36*(2), 501–511.
79. Romero, D.M.; de Molina, M.C.R.; Juárez, Á.B. Oxidative stress induced by a commercial glyphosate formulation in a tolerant strain of *Chlorella kessleri*. *Ecotoxicol. Environ. Saf.* **2011**, *74*(4), 741–747.
80. Sikorski, Ł.; Baciak, M.; Bęś, A.; Adomas, B. The effects of glyphosate-based herbicide formulations on *Lemna minor*, a non-target species. *Aquat. Toxicol*. **2019**, *209*, 70–80.
81. Smedbol, É.; Lucotte, M.; Labrecque, M.; Lepage, L.; Juneau, P. Phytoplankton growth and PSII efficiency sensitivity to a glyphosate-based herbicide (Factor 540®). *Aquat. Toxicol*. **2017**, *192*, 265–273.
82. Tsui, M.T.K.; Chu, L.M. Aquatic toxicity of glyphosate-based formulations: Comparison between different organisms and the effects of environmental factors. *Chemosphere*. **2003**, *52*, 1189–1197.
83. Iummato, M.M.; Fassiano, A.; Graziano, M.; Afonso, M.S.; Molina, M.C.R.; Juárez, A.B. Effect of glyphosate on the growth, morphology, ultrastructure and metabolism of *Scenedesmus vacuolatus*. *Ecotoxicol. Environ. Saf.* **2019**, *172*, 471–479.
84. Wagner, N.; Veith, M.; Lötters, S.; Viertel, B. Population and life-stage–specific effects of two herbicide formulations on the aquatic development of European common frogs (*Rana temporaria*). *Environ. Toxicol. Chem*. **2017**, *36*(1), 190–200.
85. Wojtaszek, B.F.; Staznik, B.; Chartrand, D.T.; Stephenson, G.R.; Thompson, D.G. Effects of Vision® herbicide on mortality, avoidance response, and growth of amphibian larvae in two forest wetlands. *Environ. Toxicol. Chem: An International Journal*. **2004**, *23*(4), 832–842.
86. Zhang, Q.; Qu, Q.; Lu, T.; Ke, M.; Zhu, Y.; Zhang, M.; Zhang, Z.; Du, B.; Pan, X.; Sun, L.; Qian, H. The combined toxicity effect of nanoplastics and glyphosate on *Microcystis aeruginosa* growth. *Environ. Pollut*. **2018**, *243*, 1106–1112.
87. Angel, B.M.; Batley, G.E.; Jarolimek, C.V.; Rogers, N.J. The impact of size on the fate and toxicity of nanoparticulate silver in aquatic systems. *Chemosphere*. **2013**, *93*(2), 359–365.
88. Asghari, S.; Johari, S.A.; Lee, J.H.; Kim, Y.S.; Jeon, Y.B.; Choi, H.J.; Yu, I.J. Toxicity of various silver nanoparticles compared to silver ions in *Daphnia magna*. *Jour. Nanobiotech.* **2012**, *10*(1), 14.
89. Bielmyer-Fraser, G.K.; Jarvis, T.A.; Lenihan, H.S.; Miller, R.J. Cellular partitioning of nanoparticulate versus dissolved metals in marine phytoplankton. *Environ. Sci. Techol.* **2014**, *48*(22), 13443–13450.
90. Burchardt, A.D.; Carvalho, R.N.; Valente, A.; Nativo, P.; Gilliland, D.; Garcìa, C.P.; Passarella, R.; Pedroni, V.; Rossi, F.; Lettieri, T. Effects of silver nanoparticles in diatom *Thalassiosira pseudonana* and *cyanobacterium Synechococcus sp. Environ. Sci. Technol.* **2012**, *46*(20), 11336–11344.
91. Blinova, I.; Niskanen, J.; Kajankari, P.; Kanarbik, L.; Käkinen, A.; Tenhu, H.; Kahru, A. Toxicity of two types of silver nanoparticles to aquatic crustaceans *Daphnia magna* and *Thamnocephalus platyurus*. *Enviro.l Sci. Poll. Resear*. **2013**, *20*(5), 3456–3463.
92. Chae, Y.J.; Pham, C.H.; Lee, J.; Bae, E.; Yi, J.; Gu, M.B. Evaluation of the toxic impact of silver nanoparticles on Japanese medaka (*Oryzias latipes*). *Aquat. Toxicol.* **2009**, *94*(4), 320–327.
93. Dewez, D.; Oukarroum, A. Silver nanoparticles toxicity effect on photosystem II photochemistry of the green alga *Chlamydomonas reinhardtii* treated in light and dark conditions. *Toxicol. Environ. Chem.* **2012**, *94*(8), 1536–1546.
94. Ellegaard-Jensen, L.; Jensen, K.A.; Johansen, A. Nano-silver induces dose-response effects on the nematode *Caenorhabditis elegans*. *Ecotoxicol. Environ. Saf.* **2012**, *80*, 216–223.
95. Gil-Allué, C.; Schirmer, K.; Tlili, A.; Gessner, M.O.; Behra, R. Silver nanoparticle effects on stream periphyton during short-term exposures. *Environ. Sci. Technol*. **2015**, *49*(2), 1165–1172.
96. Govindasamy, R.; Rahuman, A.A. Histopathological studies and oxidative stress of synthesized silver nanoparticles in Mozambique tilapia (*Oreochromis mossambicus*). *Jour. Environ. Sci*. **2012**, *24*(6), 1091–1098.
97. Griffitt, R.J.; Luo, J.; Gao, J.; Bonzongo, J.C.; Barber, D.S. Effects of particle composition and species on toxicity of metallic nanomaterials in aquatic organisms. *Environ. Toxicol. Chem.* **2008**, *27*, 1972–1978.
98. Gubbins, E.J.; Batty, L.C.; Lead, J.R. Phytotoxicity of silver nanoparticles to *Lemna minor L. Environ. Pollut*. **2011**, *159*(6), 1551–1559.
99. Hoheisel, S.M.; Diamond, S.; Mount, D. Comparison of nanosilver and ionic silver toxicity in *Daphnia magna* and *Pimephales promelas*. *Environ. Toxicol. Chem*. **2012**, *31*(11), 2557–2563.
100. Jiang, H.S.; Li, M.; Chang, F.Y.; Li, W.; Yin, L.Y. Physiological analysis of silver nanoparticles and AgNO3 toxicity to *Spirodela polyrhiza*. *Environ. Toxicol. Chem*. **2012**, *31*(8), 1880–1886.
101. Jo, H.J.; Choi, J.W.; Lee, S.H.; Hong, S.W. Acute toxicity of Ag and CuO nanoparticle suspensions against *Daphnia magna*: The importance of their dissolved fraction varying with preparation methods. *J. Hazard. Mater.* **2012**, *22–228*, 301–308.

102. Kashiwada, S.; Ariza, M.E.; Kawaguchi, T.; Nakagame, Y.; Jayasinghe, B, S.; Gärtner K.; Kagami, Y.; Sabo-Attwood, S.; Fergunson, P.L.; Chandler, G.T. Silver nanocolloids disrupt medaka embryogenesis through vital gene expressions. *Environ. Sci. Technol.* **2012**, *46*, 6278–6287.
103. Kennedy, A.J.; Hull, M.S.; Bednar, A.J.; Goss, J.D.; Gunter, J.C.; Bouldin, J.L.; Vikesland, P.J.; Steevens, J.A. Fractionating nanosilver: Importance for determining toxicity to aquatic test organisms. *Environ. Sci. Tech.* **2010**, *44*(24), 9571–9577.
104. Kennedy, A.J.; Chappell, M.A.; Bednar, A.J.; Ryan, A.C.; Laird, J.G.; Stanley, J.K.; Steevens, J.A. Impact of organic carbon on the stability and toxicity of fresh and stored silver nanoparticles. *Environ. Sci. Technol.* **2012**, *46*, 10772–10780.
105. Kim, J.; Kim, S.; Lee, S. Differentiation of the toxicities of silver nanoparticles and silver ions to the Japanese medaka (*Oryzias latipes*) and the cladoceran *Daphnia magna*. *Nanotoxicology*. **2011**, 5(2), 208–214.
106. Kwok, K.W.; Auffan, M.; Badireddy, A.R.; Nelson, C.M.; Wiesner, M.R.; Chilkoti, A.; Hinton, D.E. Uptake of silver nanoparticles and toxicity to early life stages of Japanese medaka (*Oryzias latipes*): Effect of coating materials. *Aquat. Toxicol*. **2012**, *120*, 59–66.
107. Laban, G.; Nies, L.F.; Turco, R.F.; Bickham, J.W.; Sepúlveda, M.S. The effects of silver nanoparticles on fathead minnow (*Pimephales promelas*) embryos. *Ecotoxicology*. **2010**, *19*(1), 185–195.
108. Lee, Y.J.; Kim, J.; Oh, J.; Bae, S.; Lee, S.; Hong, I.S.; Kim, S.H. Ion-release kinetics and ecotoxicity effects of silver nanoparticles. *Environ. Toxicol. Chem.* **2012**, *31*(1), 155–159.
109. McLaughlin, J.; Bonzongo, J-C. J. Effects of natural water chemistry on nanosilver behavior and toxicity to *Ceriodaphnia dubia* and *Pseudokirchneriella subcapitata*. *Environ. Toxicol. Chem.* **2012**, *31*, 168–175.
110. Navarro, E.; Piccapietra, F.; Wagner, B.; Marconi, F.; Kaegi, R.; Odzak, N.; Sigg, L.; Behra, R. Toxicity of silver nanoparticles to *Chlamydomonas reinhardtii*. *Environ. Sci. Techol.* **2008**, *42*(23), 8959–8964.
111. Pérez, S., Moreno-Garrido, I., Capitán-Valley, L.F., Lapresta-Fernández, A., Lubián, L.M., Blasco, J., 2014. *Toxicity of silver and glod-silver alloy nanoparticles in the marine and freshawater microalgae*. In: Abstracts of the IV Congress of Marine Sciences. Las Palmas de Gran Canaria, Spain, June 11th to 13th, 2014.
112. Piccapietra, F.; Allué, C.G.; Sigg, L.; Behra, R. Intracellular silver accumulation in Chlamydomonas reinhardtii upon exposure to carbonate coated silver nanoparticles and silver nitrate. *Environ. Sci. Techol*. **2012**, *46*(13), 7390–7397.
113. Poynton, H.C.; Lazorchak, J.M.; Impelliteri, C.A.; Blalock, B.J.; Rogers, K.; Allen H. J.; Loguinov, A.; Heckman, J.L.; Govindasmawy, S. Toxicogenomic responses of nanotoxicity in *Daphnia magna* exposed to silver nitrate and coated silver nanoparticles. *Environ. Sci. Technol.* **2012**, *46*, 6288–6296.
114. Ribeiro, F.; Gallego-Urrea, J.A.; Jurkschat, K.; Crossley, A.; Hassellöv, M.; Taylor, C.; Loureiro, S. Silver nanoparticles and silver nitrate induce high toxicity to Pseudokirchneriella subcapitata, *Daphnia magna* and *Danio rerio*. *Science of the Total Environment*. **2014**, *466*, 232–241.
115. Sendra, M.; Yeste, M.P.; Gatica, J.M.; Moreno-Garrido, I.; Blasco, J. Direct and indirect effects of silver nanoparticles on freshwater and marine microalgae (*Chlamydomonas reinhardtii* and *Phaeodactylum tricornutum*). *Chemosphere*. **2017**, *179*, 279–289.
116. Sendra, M.; Blasco, J.; Araújo, C.V. Is the cell wall of marine phytoplankton a protective barrier or a nanoparticle interaction site? Toxicological responses of *Chlorella autotrophica* and *Dunaliella salina* to Ag and CeO2 nanoparticles. *Ecol indics*. **2018**, *95*, 1053–1067.
117. Tuominen, M.; Sillanp_a_a, M.; Schultz, E. Toxicity and stability of silver nanoparticles to the green alga *Pseudokirchneriella subcapitata* in boreal freshwater samples and growth alga. *Nanomater. Environ*. **2013**, 48–57.
118. Wang, Z.; Chen, J.; Li, X.; Shao, J.; Peijnenburg, W.J.G.M. Aquatic toxicity of nanosilver colloids to different trophic organisms: Contributions of particles and free silver ion. *Environ. Toxicol. Chem.* **2012**, *31*, 2408–2413.
119. Wu, Y.; Zhou, Q. Silver nanoparticles cause oxidative damage and histological changes in medaka (*Oryzias latipes*) after 14 days of exposure. *Environ. Toxicol. Chem*. **2013**, *32*(1), 165–173.
120. Zhao, C.M.; Wang, W.X. Comparison of acute and chronic toxicity of silver nanoparticles and silver nitrate to *Daphnia magna*. *Environ. Toxicol. Chem.* **2011**, *30*, 885– 892.
